# Permanently online and permanently connected: Development and validation of the Online Vigilance Scale

**DOI:** 10.1371/journal.pone.0205384

**Published:** 2018-10-25

**Authors:** Leonard Reinecke, Christoph Klimmt, Adrian Meier, Sabine Reich, Dorothée Hefner, Katharina Knop-Huelss, Diana Rieger, Peter Vorderer

**Affiliations:** 1 Department of Communication, Johannes Gutenberg University Mainz, Mainz, Germany; 2 Department of Journalism and Communication Research, Hanover University of Music, Drama, and Media, Hanover, Germany; 3 Department of Communication Science and Media Research, Ludwig-Maximilians University Munich, Munich, Germany; 4 Institute for Media and Communication Studies, University of Mannheim, Mannheim, Germany; Geneva University, SWITZERLAND

## Abstract

Smartphones and other mobile devices have fundamentally changed patterns of Internet use in everyday life by making online access constantly available. The present paper offers a theoretical explication and empirical assessment of the concept of *online vigilance*, referring to users’ permanent cognitive orientation towards online content and communication as well as their disposition to exploit these options constantly. Based on four studies, a validated and reliable self-report measure of online vigilance was developed. In combination, the results suggest that the Online Vigilance Scale (OVS) shows a stable factor structure in various contexts and user populations and provides future work in communication, psychology, and other social sciences with a new measure of the individual cognitive orientation towards ubiquitous online communication.

## Introduction

Around the world, the proliferation of mobile Internet technologies–wireless online connections and portable devices such as smartphones–is driving fundamental changes in how people practice and think about communication in their daily lives [1]. It is only a few years ago that connecting to the Internet, using online media, and communicating with others via technology were actions that required conscious planning, mental effort, and specific arrangements, such as sitting down in front of a computer. Mobile Internet technologies and online devices have changed this situation dramatically: Being involved in mediated communication and/or maintaining availability to communicate is now almost the default situation for many people and basically taken for granted most of the day [2,3]. In contrast, *abstaining* from media use and communication access now appears to be a quite exotic action that requires unusual intentions and conscious planning and may even trigger negative affect and anxiety (e.g., [4,5]). The common assumption many users of smartphones and other mobile online devices seem to hold is that online content and communication are accessible and meaningful tools of goal attainment and need satisfaction at virtually any time and any place. They are living in a media-saturated word that enables them to be “permanently online and permanently connected” (POPC) [6–8].

In this new POPC environment, users have developed specific routines and cognitive structures concerning their mobile online device(s), their communication relationships, and the role of receiving and sending information in the course of their daily lives [9]. They can rely on their ‘always-on’ equipment to solve diverse problems, manage their mood, and create a “24/7 communicative bubble” (p. 317, [10]) that continuously connects them to relevant others.

The present contribution offers a conceptual definition and develops an empirical measure of connectedness as an individual difference variable. While our current POPC communication environment affords permanent *technological* connectedness, not all users have internalized this possibility for connection *psychologically* to the same degree (i.e., not everybody is psychologically permanently connected). With reference to the psychological concept of *vigilance*, which describes “the ability to sustain attention to a task” (p. 1885, [11]) or the “psychological readiness to perceive and respond” (p. 6, [12]), we call this concept *online vigilance* [13]. Online vigilance refers to individual differences in three aspects of users’ psychology: (1) their *cognitive orientation* to permanent, ubiquitous online connectedness; (2) their chronic *attention* to and continuous integration of online-related cues and stimuli into their thinking and feeling; and (3) their *motivational disposition* to prioritize options for online communication over other (offline) behavior. These defining features of online vigilance (cognitive orientation, chronic attention, and motivational disposition to the online sphere) find their expression in three sub-dimensions of online vigilance: People who are high in online vigilance will think more often and more intensively about their personal online sphere even when they are *not* using their mobile device (*salience* of the online world). Strong online vigilance also entails readiness to react to cues received via (mobile) online communication quickly, even if this means interrupting (important) other activities (*reactibility*). The final component of online vigilance is the tendency to actively observe one’s online communication environment in parallel to ongoing offline activities (*monitoring*). The defining components of online vigilance are represented to varying degrees within its three sub-dimensions (e.g., the cognitive orientation component is particularly strongly related to the salience sub-dimension, whereas the motivational component of online vigilance corresponds particularly strongly with the reactibility sub-dimension). However, cognitive, attentional, and motivational processes are of high relevance for all three sub-dimensions, as will be discussed below.

While many people nowadays own and use mobile online devices such as smartphones, they clearly vary with regard to the extent that smartphones and mobile applications shape their daily routines [14]. Conceptualizing and measuring such variation in online vigilance is an important challenge for communication research, because (mobile) online communication and users’ cognitive, affective, and motivational stance towards it are likely to play an important role across many domains of the field. With the proposition of the concept of online vigilance and the development of an adequate empirical measure, we therefore intend to provide and advance theoretical concepts and empirical measures of constant connectedness to keep up with the psychological implications of the current trend of ‘always online’ thinking and behavior. In the following sections, we will first discuss the learning mechanisms that lead to the development of online vigilance. Subsequently, the three sub-dimensions of online vigilance–salience, reactibility, and monitoring–are explicated in detail. This is followed by a systematic comparison of the conceptual similarities and differences between online vigilance and two related concepts: Internet habits and Internet addiction. The theory section concludes with a discussion of the sources of individual differences in online vigilance to further underline the need for an empirical measure of this variable.

### Explicating online vigilance

We propose that two central learning mechanisms, *instrumental learning* and *attentional learning*, interact with the affordances of online communication and the specific need structure of individual users to shape individual differences in online vigilance.

Instrumental learning refers to “learning about action-outcome relationships” (p. 319, [15]) and is a crucial mechanism in human learning, enabling individuals to adapt their behavior in goal-consistent ways and to avoid behavior linked to undesirable outcomes. Successful instrumental learning is a function of two central factors: contiguity and contingency [15]. Contiguity refers to the temporal or spatial closeness between two related events. The briefer the temporal delay or the spatial distance between an event or action and an outcome, the faster individuals are able to make valid causality inferences [16,17]. Contiguity, however, is not a sufficient condition for instrumental learning. Successful learning of the causal relationship between two events further requires contingency, that is, the cause needs to show a sufficient temporal correlation (i.e., occur prior to the outcome at a consistent rate) with the effect. Only if an event or action possesses a detectable predictiveness of an outcome can instrumental learning occur [15,18].

We believe that the affordances of online communication and of mobile Internet use in specific provide ideal preconditions for instrumental learning. Online communication provides a plethora of different gratifications [19–21] and represents a reliable source of intrinsic need satisfaction [22,23]. Mobile Internet access in particular, makes the gratifications of online communication and information constantly and instantly available, providing rapid access to problem-solving resource in almost any situation and life domain [13]. Due to the social expectations of quick responses to online messages (e.g., [24]) and the breadth and heterogeneity of the network of friends available via social media, the gratifications of online communication, for example in the form of informational social support, can often be obtained faster and at a lower cost than in the offline context [25]. From an instrumental learning perspective, the affordances of online communication thus produce high levels of both contiguity and contingency: online communication is consistently and reliably associated with a large number of positive and instantly available outcomes, providing ideal preconditions for instrumental learning. Consequently, heavy users are likely to form a positive relationship to specific online platforms, their smartphone, and other mobile online devices, and often find them indispensable. As past experience has repeatedly and reliably linked the use of certain apps and the smartphone to desired, positive outcomes (such as success in problem solving, rewards of pleasant social interaction, or media enjoyment), users develop a motivational disposition to initiate or react to incoming online communication frequently and with high priority.

While we suggest that instrumental learning provides the foundation for the development of the motivational components of online vigilance, we propose that the cognitive facets of online vigilance emerge because of attentional learning. Attentional learning refers to the fact that the attentional prioritization of stimuli can change as a result of learning processes [26]. In other words, attention–like any other form of behavior–can be conditioned through reinforcement. Attentional bias due to attentional learning emerges as a function of two central mechanisms–learned predictiveness and learned value [26]. A large body of research demonstrates that predictive cues, that is, stimuli that are “a consistent and reliable indicator of the events that follow it” (p. 1113, [26]), receive more attention than nonpredicitive cues. Learned predictiviness thus mirrors the concept of contingency in instrumental learning: cues that are consistently followed by a relevant outcome will be associated with this outcome and receive more attention in future encounters [27]. However, the predictiveness of a cue is not the only factor influencing attentional learning. The value of a cue is a second determinant of attentional learning [26]: cues signaling outcomes with larger rewards (i.e., with higher motivational value) receive more attention than cues associated with outcomes of lower value (e.g., [28]).

Similar to the strong connections to processes of instrumental learning outlined above, the affordances of online communication also provide a fruitful basis for attentional learning. Technical connection cues such as smartphone rings, vibrations, and reminders are highly prevalent and ubiquitous in a POPC environment [9]. Reacting to such connection cues is consistently associated with social gratifications or informational rewards (e.g., [29]). In terms of attentional learning, this suggests that connection cues possess high levels both of learned predictiveness and learned value: they consistently signal the availability of highly valued outcomes. In fact, a growing number of studies demonstrate that attentional learning in the context of online communication does exists and that cues related to the online sphere, such as the mere sight of a smartphone [30], the logo of a social media application [31], or incoming notifications [32] grasp immediate attention and trigger cravings for the associated gratifications of online communication. This suggests that, as a consequence of attention learning, users experiencing consistent associations between online-related cues and reward experiences will develop chronically high levels of attention for connection cues. This constant receptiveness for online-related stimuli should also increase the general psychological salience of and cognitive orientation towards the online sphere.

Importantly, attentional learning does not exclusively result in bottom-up, automatic forms of attentional bias, but also increases the likelihood of deliberately controlled, top-down attention allocation to predictive stimuli [26]. The same applies to instrumental learning which can result in both, automatic stimulus-response reactions as well as deliberately controlled, goal-directed behavior based on the learned causal relationship between action and desired outcomes [33]. Consequently, online vigilance encompasses both goal-directed, motivated behavior and attention as well as automatic responses and attention allocation–an important distinction separating it from related concepts such as media habits (see below).

After explicating the learning mechanisms underlying the formation of online vigilance, we will now turn to a more detailed description of the different expressions of online vigilance. As discussed in the introduction, our theoretical conceptualization of online vigilance distinguishes three sub-dimensions—salience, reactibility, and monitoring—which will be explicated in the following sections.

#### Salience

Many users of mobile online devices are involved in a large amount of continuing flows of communication (such as conversation groups in messenger apps) and connected to various streams of information (such as social media, news websites, or location-based games) (e.g., [34]). Through instrumental learning, they have established practices of using their mobile device to create a “pseudo-aural space” (p. 275, [35]) that allows them to access an entire personal universe of relevant people, messages, events, and the associated gratifications. The POPC technology makes rapid and frequent switching of attention between the online world and the offline physical reality easy and convenient. Therefore, heavy mobile online users are likely to experience a perceptual blurring of their offline and online spheres. Even when their smartphone is resting in their pocket, they are likely to let their mind wander into the online environment [4]. This constant cognitive engagement with the online environment is a result of attentional learning and further increased through a high perceptual receptiveness for online-related cues. We suggest labeling this dimension of online vigilance *salience* [13]. In the present context, salience means that users who are embedded in a given situation–at work, at home, doing sports, meeting friends, waiting for somebody or something, etc.–will dedicate parts of their thoughts to their online sphere. This includes processing information they have acquired from previous mobile online use and reflecting on what is (presumably) happening in their online context while they are simultaneously facing an offline (social) situation [2]. It is noteworthy that such cognitive involvement with the online environment does not necessarily require active, conscious thinking or self-directed reflection at all times. Rather, many users are likely to have formed cognitive routines of automatic consideration of online-related issues, people, and events [9,36]. High levels of salience may thus mean both elaborate thinking about one’s online sphere and/or automatized, unconscious accessibility of online-related thoughts. In sum, the first sub-dimension of online vigilance, *salience*, reflects the degree to which users stay cognitively connected to their online sphere to which their smartphone (or other mobile online device) serves as a constantly available portal.

#### Reactibility

As discussed above, many individuals develop strong dispositions for smartphone use via instrumental learning, because they generate positive experiences [29,37] and serve as portals to many and diverse gratifications (e.g., [38]). Being ready to respond quickly to notifications, incoming messages, and communication opportunities thus means to capitalize on chances of social gratifications, such as staying in touch with online groups of friends [39], and avoiding social sanctions, like the repercussions of responding to urgent notifications ‘late’ [24]. Consequently, the routine way in which a user with high levels of online vigilance handles her or his devices is characterized by a chronic attention for and a permanent proclivity to respond to incoming cues from the online sphere. This proclivity is strong during episodes of non-usage when a notification of the device calls for a user’s (learned) attention. Likewise, it is strong in situations when a user is already attending to the smartphone and has to decide whether to prioritize responding to the displayed information (e.g., a new social media post by a friend) or dealing with the demands of the current offline situation. We label this continuous inclination to respond and to prioritize events and cues from the online sphere over the demands of the current offline environment *reactibility* [13].

#### Monitoring

Online vigilance encompasses a third dimension, which refers to people’s tendency to actively enter their online sphere on a regular basis [13]. For many users, mobile social and interpersonal media, such as WhatsApp or Facebook, have become the central channel for communication with and information from their online social sphere. Social media content is produced and circulated via ‘threads’, ‘feeds’, and ‘timelines’, and the different social media accounts represent repositories for communication incidents (postings, shared media content, etc.) originating from interactions with online friends and acquaintances. The ongoing flow of online communication archived in the social media context represents a manifestation of the recent ‘proceedings’ of the community–messages, images, videos, etc.–and provides a real-time overview of the status quo, recent developments, and ongoing interactions in the user’s online environment(s) (e.g., [40]).

Because heavy users assign great personal relevance to them and have learned that they consistently provide rewarding experiences, they will routinely *monitor* these online repositories to maintain a constantly updated knowledge about their online social sphere [29]. This form of “background listening” connects users to a constant stream of online content and messages throughout the day (p. 528 [34]). Oftentimes, episodes of just a few seconds of smartphone use suffice to complete such updates. Consequently, frequent monitoring maintains the sense of permanence in connectedness. This provides users with a sense of “perpetual contact” (p. 312, [10]) and of experientially sharing the social life of their online friends in (near) real-time [41].

### Differentiating online vigilance from related concepts

At first glance, the concept of online vigilance seems to overlap substantially with existing terms and approaches in online research. Specifically, strong involvement with the online environment has been addressed from the perspective of media *habits* (e.g., [36,42]) as well as Internet or smartphone *addiction* (e.g., [43,44]). To further sharpen the concept of online vigilance and illustrate its usefulness beyond existing perspectives, the following section will thus differentiate online vigilance from these related theoretical constructs.

#### Media habits

The concept of *habits* refers to “learned dispositions to repeat past responses” (p. 843, [45]) and is based on “automatic associations between cues and actions that form through repetition” (p. 199, [46]). Accordingly, habits develop over time when the same behavior is frequently performed under similar conditions until it is finally triggered automatically by environmental cues [47,48]. Like any other behavior, media use in general [46] and the use of online media in specific [9,36,49] can be subject to habit formation.

The process of media habit formation shows many similarities to the development of a personal disposition for online vigilance, suggesting that both concepts may be related. In fact, previous research demonstrates that some facets of online vigilance, such as the frequent checking behavior typically associated with its *monitoring* dimension [29] or the immediate response to incoming connection cues represented by the *reactibility* dimension [9,36], are often highly habitualized.

Previous conceptualizations of media habits have tended to refer to habitual media use as a largely *unconscious* or subliminal engagement with media (i.e., reduced self-observation) and (automatic) behaviors of using or attending to devices or messages [46]. While this behavior-focused perspective on media habits fits well with some facets of online vigilance (see above), other aspects, such as the salience component of online vigilance that explicitly describe the *cognitive engagement* with and attention to the online eco-system, may appear less compatible with the habit concept. Research on *mental habits* suggests, however, that habits are not restricted to overt behavior but extend to mental processes as well [50]. According to Verplanken et al. [50], cognitive processes, such as negative self-thoughts, can reach the status of a mental habit “to the degree to which such thinking occurs frequently, is initiated without awareness, and is mentally efficient” (p. 527). Furthermore, in accordance with the research on attentional learning discussed above, recent research on *attention habits*, a specific form of mental habits, suggests that reward-related stimuli can automatically capture attention, making rewarding stimuli harder to ignore [51].

In our theoretical explication of online vigilance, we have proposed that the concept encompasses both goal-directed forms of behavior and attention as well as automatic responses to connection cues and attention allocation. The research reviewed above suggests that habits may account for these automatic components of online vigilance. While this implies that the concept of online vigilance is highly *inclusive* of media habits, we propose that it goes significantly beyond the habit construct. As expressed in our concept explication, we believe, that online vigilance is not exclusively driven by situational cues triggering automatic behavior or cognition. One central implication of habit automaticity is that the triggering of habits is largely insensitive to changes in goals [52]. Consequently, habits may represent the goals initially underlying the behavior prior to habit formation. Once a habit is formed, however, the behavior is triggered by situational cues irrespective of the individual’s present goal state [45]. While we believe that processes related to online vigilance are frequently triggered automatically by connection cues, we propose that the concept of online vigilance also includes reflective and goal-direct behavior. Thinking about and monitoring the online context as well as responding to incoming online message are not necessarily or exclusively automatic processes but may be deliberative and goal-driven efforts to cope with situational needs, such as the fear of missing out [53] or the fear of ostracism [54], or to obtain positive gratifications such as mood improvement. In fact, a large body of research characterizes online media use as goal-related and driven by situationally deprived needs such as relatedness [23]. Similarly, the decision to prioritize reacting to an incoming message over other, concurrent offline tasks or interactions is not necessarily an impulsive reaction but can also involve reflective information processing. Consequently, the theoretical concept of online vigilance is not intended to replace the concept of media habits. Instead, we believe that the present research fruitfully extends previous attempts to understand the phenomenon of constant connectedness [9,36] by providing a theoretical perspective on individual differences in cognitive and behavioral orientation toward the online environment that accounts for both, deliberate and goal-driven as well as automatic and uncontrolled processes.

#### Internet addiction

With regard to its distinctiveness from other concepts, online vigilance could also be criticized as representing a mere manifestation or symptom of *Internet addiction*. Despite the large and steadily growing body of research on Internet addiction that has evolved over the last two decades, a commonly accepted definition of the concept is still missing [43]. While the operational definitions of Internet addiction also differ considerably between individual studies, a number of frequently assessed criteria or symptoms such as tolerance, withdrawal, loss of control, and negative consequences in different life domains have been established [43,55].

The concept of online vigilance shows similarities to some of the symptoms typically discussed in the context of online addiction [56]. This connection is particularly apparent in the *salience* dimension. Cognitive preoccupation with Internet use is frequently assessed as a central indicator of Internet addiction in general [43,57] and smartphone addiction in specific [44]. However, salience as a dimension of online vigilance refers to a general cognitive orientation to and attention for the online environment in everyday life, whereas cognitive preoccupation in the context of Internet addiction refers to extreme and pathological forms of salience such as “obsessive thoughts” (p. 193, [57]) or an “irresistible urge to go online” (p. 224, [58]).

Furthermore, attempts to adapt symptoms typically associated with substance abuse, such as excessive involvement, preoccupation, or tolerance, to the context of behavioral addictions such as Internet or smartphone addiction have lately come under scrutiny. Billieux et al. [59] criticize the adoption of traditional substance abuse symptoms to diagnose behavioral addiction as atheoretical and see a problematic trend in addiction research to “overpathologize everday life” and “create innovative yet absurd addictive disorders” (p. 119). Kardefelt-Winther et al. [60] support this view and underline that symptoms that may be problematic in one context may not be so in others: “preoccupations with video games are still considered harmful in a similar way to preoccupations with drugs, even though the former is a common everyday activity related to far fewer problematic consequences than the latter” (p. 1711). Rather than relying on symptoms such as tolerance or conflict that “are likely to manifest in relation to most activities that people find interesting” (p. 1711), they propose that definitions of behavioral addictions should focus exclusively on two components: 1) significant functional impairment that directly results from the behavior and 2) persistence over time (also see the working definition of behavioral addiction provided by Billieux et al. [61]).

As a trait-like variable, online-vigilance certainly shares the element of persistence over time with this definition of behavioral addiction. However, regarding the second defining feature of behavioral addiction, functional impairment, online vigilance can clearly be distinguished from Internet addiction. In fact, a large body of research has demonstrated that Internet addiction is associated with serious negative consequences of Internet use for users’ individual functioning [55,58]. Yet, while online vigilance may be associated with some negative effects of Internet use, such as digital stress [62] or procrastination [63], we propose that the concept represents a much more mundane form of involvement with the online environment that does not necessarily impair individual functioning and mental health. In contrast, we even suggest that online vigilance possesses the potential to increase well-being: by making the beneficial (social) gratifications of online communication more readily available, high levels of online vigilance may amplify the positive effects of online communication, such as mood regulation or relatedness need satisfaction [64].

Lastly, we suggest that online vigilance and Internet addiction show massive differences in terms of their prevalence. While current studies on the basis of large-scale probability samples suggest that only a small fraction of the general population of Internet users suffers from pathological forms of Internet addiction [43], online vigilance is a direct result of day-to-day usage practices and a common phenomenon that affects large numbers of Internet users [29,38,65]. Consequently, while the cognitive orientation towards the online context may *appear* addiction-like due to its ubiquity and partial automaticity [36], we propose that online vigilance and Internet addiction are clearly distinguishable concepts. Table 1 provides an overview of the key conceptual similarities and differences of online vigilance, Internet habits, and Internet addiction.

**Table 1:** Conceptual similarities and differences of online vigilance, Internet habits, and Internet addiction.

Conceptual Dimension	Online Vigilance	Internet Habits	Internet Addiction
Behavioral control	Integration of both deliberately controlled and automated behavior	Exclusive focus on automated behavior	Strong emphasis on loss of behavioral control
Pathological behavior	No	No	Yes
Effects of use	Both positive and negative	Both positive and negative	Exclusively negative; significant functional impairment
Prevalence	High	High	Low

*Note*. Conceptual features were extracted from the extant literature. Internet habits: LaRose [46], Verplanken [47]. Internet addiction: Brand et al. [55], Kardefelt-Winther et al. [60], Müller et al. [58].

### Measuring online vigilance: Why and how

We believe that considering online vigilance is important for studying communication behavior today and in the future. Online vigilance is likely to shape individual processes, dynamics, and outcomes of communication across various life domains. For instance, online vigilance may modulate the acquisition of political news via social media, the emotional condition and well-being over the course of a day, the choice of media entertainment, and the quality of interpersonal relationships [6,13].

Yet, why should we expect individual differences in online vigilance? Should not all smartphone users be equally affected by the technological affordances of a POPC environment and the learning mechanisms outline above? And if so, do we really need a measure of online vigilance? Following a differential susceptibility perspective on media effects [66], we believe it is highly plausible that there is substantial variation in people’s dispositional online vigilance. As outline above, the reinforcing effect of the gratifications of online media and smartphone use is a crucial precondition both for functional learning as well as attentional learning–the two underlying mechanisms proposed to drive the acquisition of high levels of online vigilance. However, the gratifications produced by the affordance of online communication do not possess the same subjective value for all users alike. One central factor of reinforcement for constant connectedness, for example, is perceived social pressure to be constantly available [62]. Yet, not all users are equally affected by social pressure. In a representative survey by Reinecke et al. [62], perceived social pressure to be constantly available was negatively correlated with age. Furthermore, in the group of older users, social pressure was not a significant predictor of the number of sent and received messages, whereas younger users increased their usage intensity as a reaction to social pressure. Apparently, reducing social pressure via increased engagement in online communication had a lower subjective value for older users, resulting in a weaker reinforcement effect on online activity. Differences in gratifications received through online interactions are also demonstrated by the results of a study by Ellison, Steinfield, and Lampe [67]. They found that users with lower levels of self-esteem profited more strongly from Facebook use in terms of increased social capital than users high in self-esteem. For user with low self-esteem, Facebook use should thus have a higher instrumental value than for those with high levels of self-esteem. Finally, the differential value of social media gratifications is also supported by results of a study by Masur, Reinecke, Ziegele, and Quiring [68] that reveals a significant effect of offline need satisfaction on the intensity of social media use. In their study, impaired need satisfaction in the offline domain was associated with more intensive motives for Facebook use, resulting in a more excessive use of the social network. Apparently, a lack of need satisfaction in the offline domain made the gratifications of online interaction more valuable and desirable for the respective users. In combination, these three examples demonstrate that the psychological development of online vigilance needs to be conceptualized as an interaction of the affordances of online communication and individual user characteristics: the more strongly the gratifications offered by Internet and smartphone use correspond with the traits and needs structure of individual users, the faster and more intensive the learning processes driving the development of online vigilance should be.

Hence, it is of central importance to be able to measure and account for individual differences in online vigilance when conducting research in online communication behavior and effects. The central empirical aim of the present study was thus to develop a valid and reliable measurement instrument, whereas the exploration of the underlying processes leading to individual differences remains an important task for future research. A series of four studies was conducted to develop and validate the *Online Vigilance Scale*. Study 1 provides a first exploratory test of the factor structure of the scale, which is replicated in Study 2 with confirmatory factor analysis. Study 3 tests the temporal stability of the Online Vigilance Scale and provides first evidence of the convergent and discriminant validity of the scale. Finally, Study 4 addresses the relationship between trait and state online vigilance by testing the scale’s ability to explain variance in situation-level online vigilance.

## Study 1: Item selection and exploratory factor analysis

The main aim of Study 1 was to develop an initial item pool measuring the three dimensions of online vigilance (salience, reactibility, and monitoring) and to select items for our Online Vigilance Scale (OVS) based on an empirical exploration of the psychometric quality of the developed items. We conceptualize and measure online vigilance as a channel-independent construct. That is, online vigilance does not require individuals to use specific online services (e.g., Facebook, WhatsApp) or devices (e.g., smartphone, tablet, desktop computer). This way, our measure focuses on the cognitive, attentional, and motivational dimensions of online vigilance without confounding these processes with the specific affordances of communication technology or content providers. This bears the benefit that the scale will still be applicable in future technological environments, as both the platforms and the technical devices used to access online content are likely to change continuously. However, to explore the factor structure and reliability of our newly developed measure in a population of users that are particularly likely to show high levels of online vigilance in the context of the *current* online usage practices, Study 1 used a sample of smartphone users for item selection.

### Method

#### Item construction

An initial pool of 27 items was developed to measure salience (e.g., “My thoughts often drift to online content”), reactibility (e.g., “When I receive an online message, I immediately give it my full attention”), and monitoring (e.g., “I constantly monitor what is happening online presently”) based on the theoretical explication presented above as well as qualitative interviews with six Internet users (3 females, 3 males, *M*_age_ = 26.5 years; *SD* = 1.64). The initial item pool included nine items for each of the three facets of online vigilance. All items had a five-point Likert-scale ranging from 1 “does not apply at all” to 5 “fully applies”.

#### Sample and procedure

A total of *N* = 229 German smartphone users (53.3% female) between the age of 14 and 67 (*M* = 34.31; *SD* = 12.04) were recruited via a commercial online access panel operated by the market research company respondi AG. Participants were informed about (1) the strictly scientific purpose of the research, (2) the expected duration and procedures, (3) their right to decline to participate and to withdraw from the research once participation has begun, (4) the confidentiality of their responses, and (5) whom to contact for debriefing or any questions concerning the study. After reading the study description including the aforementioned information, informed consent was provided by all participants by clicking the “start” button to access the online survey. To protect the privacy of our participants, no personally identifiable information were stored together with the collected survey data. The commercial market research company respondi that collected the data for Study 1 and Study 2 is certified in accordance with ISO norm 26362 and follows the ICC/ESOMAR International Code on Market, Opinion and Social Research. With regard to the participation of minors, the required consent of a parent or authorized guardian was obtained by the executing market research firm respondi, as these participants were recruited from the company’s pool of registered access panel members. For these individuals, the company ensures the principles of parent/guardian consent as part of the panel registration process.

Participants responded to an online survey that included questions regarding the socioeconomic background of the participants, their general Internet use, as well as the 27 items of the initial item pool. The participants used the Internet for an average duration of *M* = 217.63 minutes (*SD* = 158.97) per day and the great majority reported using the Internet at least several times per day (96.9%). Furthermore, the majority of respondents (79.5%) reported that they carry their smartphone with them “at all times”, and 84.7% of the participants used mobile Internet access for at least 30 minutes per day.

### Results

Exploratory principal axis factor analysis (PAF) computed in SPSS 23 was used to test the factor structure of the 27 developed items. With a Kaiser-Meyer-Olkin (KMO) score of .945, the items showed high sampling adequacy for factor analysis. As we did not expect the three sub-dimensions of online vigilance to be independent from each other, we used Promax rotation (Kappa = 4) to optimize the factor solution. The factor analysis yielded four factors with an eigenvalue > 1. The four factors explained 58.40% of the item variance. While the first three factors (Eigenvalues > 1.7) clearly represented the three sub-dimensions of online vigilance, the fourth factor (Eigenvalue = 1.07) only explained 2.02% of the item variance and showed relatively low factor loadings (< .62). Two reverse-coded items from the reactibility subscale showed the strongest factor loadings on the fourth factor, suggesting that the factor represented a methodological artefact rather than an interpretable latent construct. After the two items were removed, a second PAF yielded a three-factor solution matching the three online vigilance sub-dimensions and explaining 58.33% of the item variance. Although all remaining items loaded on their designated factor, numerous items showed low factor loadings. Furthermore, the number of items per subscale range from 7 (reactivity) to 9 (salience). To create a parsimonious and balanced measure of online vigilance that can flexibly be used in varying survey contexts, we aimed at a final number of 4 items per subscale. For this purpose, the four items showing the highest factor loadings and/or the best theoretical match with their respective facet of online vigilance were retained for the final version of the Online Vigilance Scale. A final PAF with the remaining 12 items produced three factors with eigenvalues > 1, explaining 67.14% of the variance. All items showed substantial loadings on their respective factors (≥ .59) and small cross-loadings to the other factors (≤ .20). Furthermore, all three subscales possessed high internal consistencies (Salience: Cronbach’s *α* = .91; Reactibility: *α* = .83; Monitoring: *α* = .91). Finally, confirming our expectations, all three subscales showed significant positive correlations with each other. The salience subscale significantly correlated both with the reactibility (*r* = .516, *p* < .01) and the monitoring subscale (*r* = .663, *p* < .01) which also correlated positively with each other (*r* = .551, *p* < .01). A list of all items selected for the final version of the Online Vigilance Scale as well as their factor loadings is presented in Table 2.

**Table 2:** Results of the exploratory factor analysis (Study 1) of the Online Vigilance Scale items.

Items	Salience	Reactibility	Monitoring
SA1: My thoughts often drift to online content.	**.94**	-.06	-.05
SA2: I have a hard time disengaging mentally from online content.	**.85**	.01	-.03
SA3: Even when I am in a conversation with other people, I often think about what is happening online right now in the back of my mind.	**.75**	.07	.07
SA4: Often online content occupies my thoughts, even as I am dealing with other things.	**.75**	.04	.10
RE1: When I receive an online message, my thoughts drift there immediately.	.03	**.90**	-.16
RE2: When I receive an online message, it triggers an impulse in me to check it right away.	-.05	**.75**	.05
RE3: When I receive an online message, I immediately attend to it, even if I am engaged in other things at that moment.	.05	**.62**	.14
RE4: When I receive an online message, I immediately give it my full attention.	.01	**.59**	.13
MO1: I constantly monitor what is happening online.	.02	-.05	**.91**
MO2: I often feel the urge to make sure I know what is happening online.	.04	.03	**.85**
MO3: I often start certain online applications so I don’t miss out on any news.	-.10	.05	**.86**
MO4: I always keep an eye on what is happening online at the moment.	.20	-.01	**.66**
Cronbach’s Alpha	.91	.83	.91
Scale Mean (SD)	2.16 (.95)	3.31 (.82)	2.93 (1.03)

*Note*. Factor loadings > .50 are in bold. The online survey in Study 1 was conducted in German. The English items presented in Table 1 were translated from and back-translated to German by a professional translator.

### Discussion

The results of Study 1 provide preliminary empirical support for the proposed theoretical concept of online vigilance. The factor structure of the developed item pool clearly matches the three facets of online vigilance (i.e., salience, reactibility, and monitoring) developed in the theoretical explication. Furthermore, the substantial correlations between the three factors suggest that the subscales do indeed measure different facets of the higher-order latent construct of online vigilance. Finally, the factor loadings of the selected items and the high internal consistencies of the three subscales indicate that the 12 items of the newly developed Online Vigilance Scale provide a reliable empirical measure of the POPC mindset. Study 1 is limited, however, by several factors. First, although the use of a sample of smartphone users with a strong affinity for frequent Internet use seems reasonable to provide a first exploration of the phenomenon of online vigilance, it remains unclear whether the theoretical construct, its sub-dimensions, and the factor structure of the Online Vigilance Scale can also be replicated in the general population of Internet users. Furthermore, while Study 1 provided an initial test of the reliability of the Online Vigilance Scale, its construct validity remains unclear.

## Study 2: Confirmatory factor analysis

Study 2 aimed at extending the findings of Study 1 by confirming the factor structure of the Online Vigilance Scale in an independent sample of general Internet users. Additionally, Study 2 aimed at a first exploration of the relationship between the three sub-dimensions of online vigilance and different indicators of Internet use behavior. We expected to find significant positive correlations between the three sub-dimensions of online vigilance, general and mobile Internet use, the use of different forms of online-communication (e.g., email, social media, and instant messenger use), as well as the use of different forms of online content (e.g., information, entertainment, etc.). As online vigilance represents the individual tendency to think about, respond to, or check for online content irrespective of other, potentially conflicting primary activities, we also anticipated to find a positive correlation between our Online Vigilance Scale and measures of Internet multitasking, that is, the concurrent use of online services and other activities (e.g., [69]).

### Method

#### Sample and procedure

A stratified sample of 1,024 German Internet users (51.7% male) between the age of 18 and 82 (*M* = 44.23; *SD* = 14.55) was recruited via a commercial online access panel operated by the market research company respondi AG. Informed consent was obtained with the same procedure used in Study 1. The sample is representative for the general population of Internet users in Germany in terms of age, gender, educational level, and occupational status. The majority of participants (92.5%) reported to use the Internet for one hour or more per day and 81% had mobile Internet access.

#### Measures

The 12 items of the *Online Vigilance Scale (OVS)* developed in Study 1 were also assessed in Study 2. All three subscales showed high internal consistencies (Salience: *α* = .91, *M* = 1.73, *SD* = .88; Reactibility: *α* = .87, *M* = 2.45, *SD* = .96; Monitoring: *α* = .90, M = 2.13, SD = 1.00). Participants’ daily general *Internet use* as well as their daily use of mobile Internet access was measured on a 9-point scale ranging from 1 “never” to 9 “more than 8 hours”. Furthermore, the use of different forms of online communication (i.e., email, social network sites, messenger apps, and microblogging) as well as different forms of online content (i.e., searching information, online news sites, online video platforms, and online radio and music platforms) was measured on a 5-point scale ranging from 1 “never” to 5 “very frequently”. To assess *Internet multitasking* participants indicated on a five-point scale from 0 “never” to 4 “very frequently” how often they use the Internet while they simultaneously a) use other media, b) should be working, c) are in a conversation with other persons, d) are having a meal, e) go out with their friends, and f) are in an intimate situation with their romantic partner. The scale showed a satisfactory internal consistency (Cronbach’s *α* = .79).

### Results

**Fig 1:**
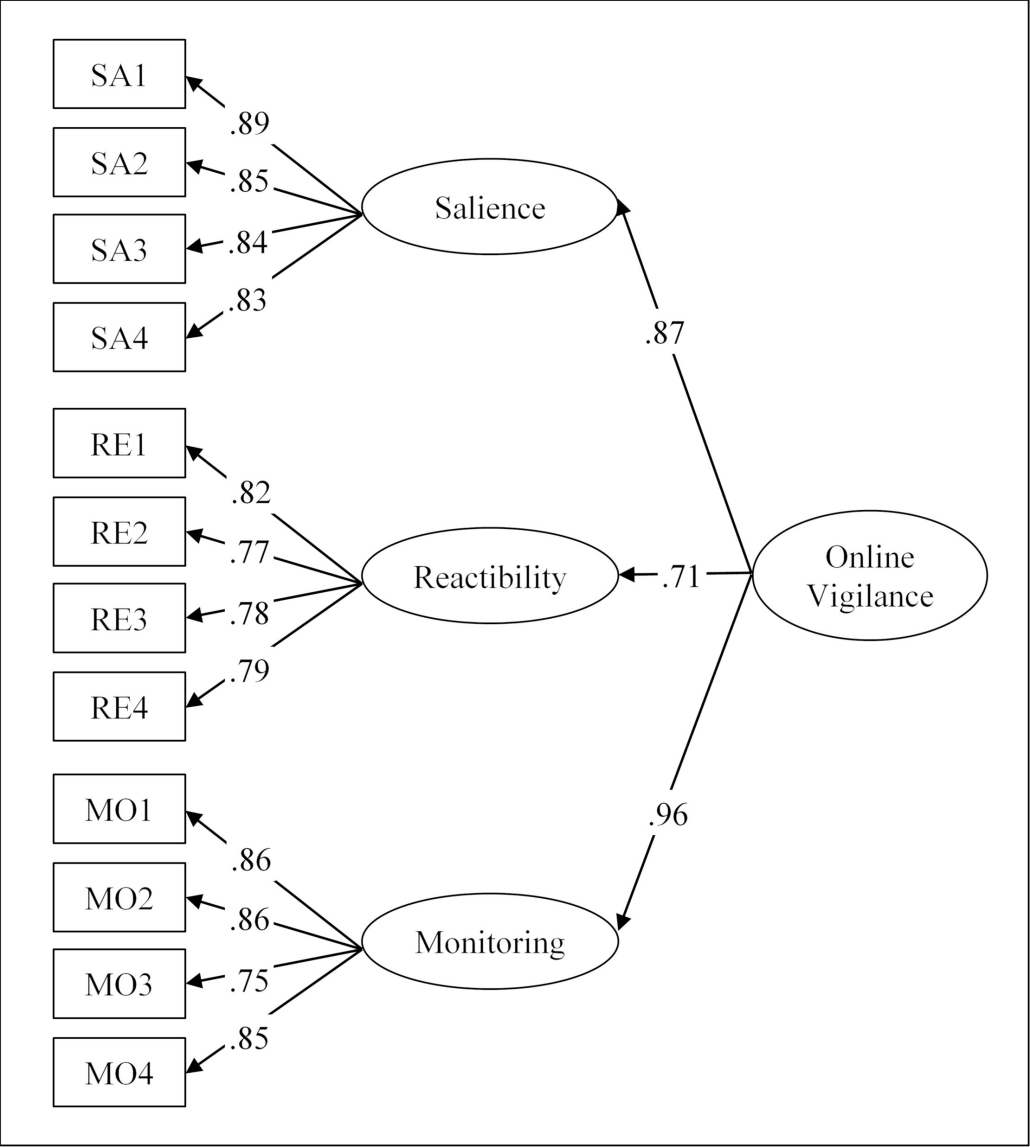
Confirmatory factor analysis of the Online Vigilance Scale. Based on data from N = 1,024 participants (Study 2), *χ*^2^(51) = 195.69, *p* < .001, CMIN/*df* = 3.84, CFI = .983, RMSEA = .053, 90% CI = [.045, .061], and SRMR = .024. Scores in the figure represent standardized path coefficients significant at *p* < .001.

A confirmatory factor analysis of the 12 items of the Online Vigilance Scale was computed using the AMOS 23 software packet and the maximum likelihood (ML) method. For this purpose a *second-order model* was computed in which the three sub-dimensions of salience, reactibility, and monitoring were modeled as separate latent variables loading on a second-order latent factor representing the higher-order concept of online vigilance (see Fig 1). As recommended by Hu and Bentler [70], model fit was estimated with a combination of three fit indices: the comparative fit index (CFI), the root mean square error of approximation (RMSEA), and the standardized root mean square of residuals (SRMR).

With *χ*^2^(51) = 195.69, *p* < .001, CMIN/*df* = 3.84, CFI = .983, RMSEA = .053, 90% CI = [.045, .061], and SRMR = .024, the second-order model fit the data well. Assessment of normality demonstrated that the data significantly deviated from multivariate normality (Mardia’s normalized estimate of multivariate kurtosis = 69.57). As recommended for the analysis of nonnormal data [71], all paths were thus additionally tested using bootstrapping. Ninety-five percent bias-corrected confidence intervals were computed for all parameters reported in Fig 1 based on 5,000 bootstrap samples with replacement. All significant statistical relationships reported below were confirmed with the bootstrap method.

All items showed significant and high loadings on their designated sub-dimensions of online vigilance (all ≥ .75, see Fig 1). Furthermore, the three sub-dimensions (salience, reactibility, and monitoring) loaded significantly and strongly (all loadings ≥ .71) on the higher-order factor of online vigilance. The confirmatory factor analysis thus replicates the results of the exploratory analysis performed in Study 1 and suggests that all 12 items show stable loadings on their respective subscales. The fact that the three sub-dimensions of online vigilance form a second-order factor further suggests that the Online Vigilance Scale can be used both to create a total score of the higher-order concept of online vigilance by combining all 12 items or, alternatively, to create separate scores for the three sub-dimensions of salience, reactibility, and monitoring.

In a further step, the factor loadings produced by the CFA were replicated using Bayesian estimation. The use of items with a Likert-type response format, such as those of the Online Vigilance Scale, in parametric tests is subject to ongoing debate. While some researchers suggest that Likert-type items should be treated as rank-ordered categorical data [72], others defend this common practice [73,74]. Both empirical research as well as simulation studies suggest that most parametric statistical tests, including the Pearson correlation coefficient [74,75] as well as exploratory and confirmatory factor analysis [76,77], are relatively robust against the use of ordinal data. However, to increase the confidence in our results and to address any potential problems that may arise from the Likert-type response format of our items, we also computed the factor loadings of all items based on Bayesian parameter estimates as recommended by Byrne [71] for the use of rank-ordered categorical data in CFA. Bayesian estimation was performed in Amos 23 based on Markov chain Monte Carlo (MCMC) techniques and a diffuse prior distribution. The posterior distribution reached sufficient convergence after 500 burn-in samples and 235,500 analysis samples with a potential scale reduction of $\hat{R}$ ≤ 1.0013 for all estimates [78]. Posterior means were used as point estimates for the unstandardized factor loadings of all items on their respective factor [71]. Ninety-five-percent Bayesian credibility intervals suggest that all factor loadings deviate from zero. Point estimates of all unstandardized factor loadings based on ML and Bayesian estimation can be found in Table 3. All Bayesian point estimates are identical to or closely resemble ML estimates. The Bayesian analyses thus fully replicated the results of the ML-based CFA.

**Table 3:** Comparison of Maximum Likelihood (ML) and Bayesian estimates of unstandardized factor loadings (regression weight) of the Online Vigilance Scale Items (Study 2).

	ML Estimates			Bayesian Estimates		
Path	*b*	*SE*	*p*	Posterior Mean	95% Lower Bound	95% Upper Bound
SA1 <—Salience	1.000	-	-	1.000	-	-
SA2 <—Salience	.912	.025	< .001	.913	.866	.963
SA3 <—Salience	.917	.025	< .001	.917	.868	.970
SA4 <—Salience	.956	.027	< .001	.956	.903	1.013
RE1 <—Reactibility	1.000	-	-	1.000	-	-
RE2 <—Reactibility	1.020	.039	< .001	1.020	.945	1.102
RE3 <—Reactibility	.930	.035	< .001	.931	.861	1.003
RE4 <—Reactibility	.938	.035	< .001	.939	.870	1.012
MO1 <—Monitoring	1.000	-	-	1.000	-	-
MO2 <—Monitoring	1.063	.030	< .001	1.063	1.006	1.123
MO3 <—Monitoring	.961	.034	< .001	.962	.901	1.027
MO4 <—Monitoring	1.028	.030	< .001	1.028	.972	1.086

In a last step, we explored the relationship between the three subscales of the Online Vigilance Scale and different measures of Internet use. All three subscales showed the expected patterns of correlations with the selected criterion variables (see Table 4). Online vigilance was significantly related to all of our measures of Internet use and showed particularly strong connections with mobile Internet use, Internet multitasking, and social media use.

**Table 4:** Zero-order correlations between the sub-dimensions of the Online Vigilance Scale and indicators of Internet use (Study 2).

Criterion variable	Salience	Reactibility	Monitoring
Modes of Internet Use:			
General Internet Use	.22**	.20**	.30**
Mobile Internet Use	.34**	.30**	.40**
Internet Multitasking	.56**	.45**	.60**
Use of Online Communication:			
Email	.07*	.10**	.12**
Messenger Apps (e.g., WhatsApp)	.25**	.27**	.35**
Social Network Sites (e.g., Facebook)	.24**	.19**	.37**
Microblogging (e.g., Twitter)	.34**	.23**	.42**
Use of Online Content:			
Searching Information	.10**	.18**	.16**
Online News	.18**	.22**	.29**
Online Video	.27**	.24**	.37**
Online Music and Radio	.29**	.25**	.34**

Note.

* p < .05

** p < .01.

### Discussion

Study 2 confirms the factor structure of the Online Vigilance Scale in a large, independent sample of Internet users. It thus underlines the factorial stability of the scale as well as the internal consistency and reliability of its subscales. Furthermore, Study 2 demonstrates the applicability of the Online Vigilance Scale beyond the sub-population of smartphones users and shows that the phenomenon of online vigilance extends to the general population of Internet users.

Moreover, Study 2 provides preliminary evidence for the validity of the Online Vigilance Scale. As predicted, the scale showed significant positive correlations with POPC behavior (Internet and social media use, Internet multitasking). The findings provide further insights into the nature of online vigilance. The Online Vigilance Scale showed similar correlations with social media use as well as more information- and entertainment-related forms of Internet use. This suggests that while previous research has primarily focused on social norms and social gratifications of Internet use as central triggers of online behavior [9,38], the individual disposition for online vigilance also extends to other forms of online content. Furthermore, our data suggest that online vigilance is not only related to how intensively online content and communication is used, but also how Internet use is integrated into and interwoven with the everyday life of Internet users: The high correlations between the Online Vigilance Scale and Internet multitasking demonstrate that users with high levels of online vigilance are constantly willing to “make room” for Internet use. This strongly supports the notion of online vigilance as a strong and permanent occupation with and motivational prioritization of Internet use irrespective of primary activities and the demands of the offline context.

## Study 3: Construct validity and test-retest reliability

While Study 1 and Study 2 provide considerable support for our general conceptualization of online vigilance as well as the reliability of our newly developed measure, the statistical relationship between the three facets of online vigilance and related constructs as well as the temporal stability of online vigilance is an open question. As discussed above, the construct of online vigilance is related to but distinct from the concept of media habits. Study 3 thus tested the statistical relationship between online vigilance and Internet and smartphone habits to provide first evidence of the discriminant validity of the Online Vigilance Scale. Furthermore, previous research provides preliminary evidence of potential drivers and outcomes of online vigilance. A number of studies have identified the fear of missing out on gratifying social events and experiences (FOMO) as a central driver of the use of and cognitive preoccupation with social media and online communication (e.g., [53,62]). A growing body of research also suggests that a POPC lifestyle may result in substantial levels of ‘digital stress’ (e.g., [62,79]). In Study 3, we thus explored the relationship between online vigilance, FOMO, and stress and expected to find positive correlations between the three variables as an indicator of convergent validity of the Online Vigilance Scale. Finally, Study 3 also aimed at providing initial evidence of the conceptual differences between online vigilance and Internet addiction. In contrast to Internet addiction, which is defined by significant functional impairment resulting from pathological usage patterns, online vigilance should also have a positive, functional side and the potential to show beneficial effects on users’ well-being (see Table 1). Specifically, being vigilant towards one’s online platforms should facilitate mediated social interaction and connectedness to loved ones and peers in everyday life [22,23,41], whereas Internet addiction is frequently associated with social isolation and lower quality of social interactions [58]. Accordingly, we would expect individuals with higher online vigilance to also report higher satisfaction of their need for relatedness in online communication [80].

Internet use and online communication are likely to show considerable day-level fluctuations depending on numerous boundary conditions, such as number of received messages, day-specific time constraints, or situational presence of connection cues. If our notion of online vigilance as a personal disposition were correct, however, we would expect to find considerable intra-individual temporal stability in our measure of person-level online vigilance despite these day-level fluctuations. Besides these conceptual considerations, a high temporal consistency of our measure also has crucial methodological implications and is a central indicator of measurement reliability. The second aim of Study 3 was thus to evaluate the test-retest reliability of the Online Vigilance Scale.

### Method

#### Sample and procedure

A total of *N* = 532 student smartphone users (65.4% female, *M*_age_ = 22.58 years, *SD* = 2.44) participated in an online diary study. Participants were invited to the study by 84 student recruiters enrolled in the communication program at a large university in Germany. The recruiters distributed the invitation to participate in the online diary study in exchange for course credit. Informed consent was obtained with the same procedure used in Study 1 and Study 2. The study consisted of two parts: A baseline screening questionnaire and a series of online diary surveys that was sent to participants via email on five consecutive workdays. Participants were instructed to fill out the online diaries before going to bed. The baseline questionnaire assessed all person-level constructs used to test construct validity (i.e., FOMO, smartphone habit strength, social networking site (SNS) habit strength, and relatedness satisfaction in online communication), whereas the daily diaries assessed a number of Internet use variables as well as day-level fluctuations in hedonic and eudaimonic well-being, specifically, perceived stress. Results regarding the main study variables are presented elsewhere. Additionally, participants responded to the 12 items of the Online Vigilance Scale (OVS) on the first (T1) and fifth day (T2) of the diary study. The test-retest reliability of the scale is tested based on these two points of measurement.

#### Measures

Online vigilance was assessed with the same scale as in Study 2. All three subscales of the Online Vigilance Scale showed high levels of internal consistency at both points of measurement and mean levels comparable to the previous two studies (Salience: *M*_T1_ = 1.89, *SD*_T1_ = 0.81, *M*_T2_ = 1.79, *SD*_T2_ = 0.82, *α*_T1_ = .86, *α*_T2_ = .89; Reactibility: *M*_T1_ = 2.86, *SD*_T1_ = 0.92, *M*_T2_ = 2.66, *SD*_T2_ = 0.96, *α*_T1_ = .86, *α*_T2_ = .90; Monitoring: *M*_T1_ = 2.47, *SD*_T1_ = 0.96, *M*_T2_ = 2.30, *SD*_T2_ = 0.97, *α*_T1_ = .89, *α*_T2_ = .90).

The *fear of missing out* (FOMO) was measured with a four-item short-form of the Fear of Missing Out scale (FOMOs) [53]. We selected the first four items of the ten-item full scale ([53], Appendix A), since we required an economical short-scale and these four items had the highest face-validity. The short-form (*M* = 2.51, *SD* = 1.01) was measured on a 5-point scale ranging from 1 “does not apply at all” to 5 “fully applies” and showed adequate internal consistency (α = .83).

*Smartphone habit strength* and *SNS habit strength* were each measured with the four items of the Self-Report Behavioral Automaticity Index (SRBAI) [81]. The two measures were assessed with 7-point scales ranging from 1 “does not apply at all” to 7 “fully applies”. Both smartphone habit strength (*M* = 4.58, *SD* = 1.47) and SNS habit strength (*M* = 3.67, *SD* = 1.80) showed high internal consistencies (α_smartphone_ = .88, α_SNS_ = .92). In the baseline questionnaire, the two scales were placed between several unrelated scales to reduce collinearity. They were moderately correlated (*r* = .55, *p* < .001).

*Relatedness need satisfaction in online communication* was measured with three items from the Balanced Measure of Psychological Needs (BMPN) [82]. The scale was adapted to measure relatedness need satisfaction in the context of interpersonal online communication. Items were assessed on a 5-point scale ranging from 1 “does not apply at all” to 5 “fully applies”. Participants were asked to rate the following three statements: “When I communicate online with others (e.g., via WhatsApp, Snapchat, or Facebook)…” (1) “…I feel a sense of contact with people who care for me, and whom I care for.”, (2) “…I feel close and connected with other people who are important to me.”, and (3) “…I feel a strong sense of intimacy with the people I communicate with.” The scale (*M* = 3.38, *SD* = 0.84) showed adequate internal consistency (α = .77).

Finally, *perceived stress* was measured each day in the online diary with three items from the Perceived Stress Scale [83]. We used this short-form of the scale to economically assess perceived stress at day-level. The three items were “Today, how strongly did you feel that you were unable to control the important things in your life”, “Today, how strongly did you feel nervous and ‘stressed’?”, and “Today, how strongly did you feel difficulties were piling up so high that you could not overcome them?”. Items were rated on a 5-point scale from 1 “not at all” to 5 “very strongly”. Internal consistencies ranged from α = .76 to α = .81 across the five days. Since we were only interested in the relationships at person-level for construct validation, we created mean aggregates of participants’ responses to the three items from all days, with no imputation of missing values from days without responses. These aggregated item scores are used to estimate perceived stress at person-level (*M* = 2.47, *SD* = 0.82).

While all scales used in Study 3 have been validated in English, only the Perceived Stress Scale and the Balanced Measure of Psychological Needs have been validated for the German language. The FOMO scale and the habit scales used in this study were translated to German and translations were cross-checked by the research team.

### Results

**Fig 2:**
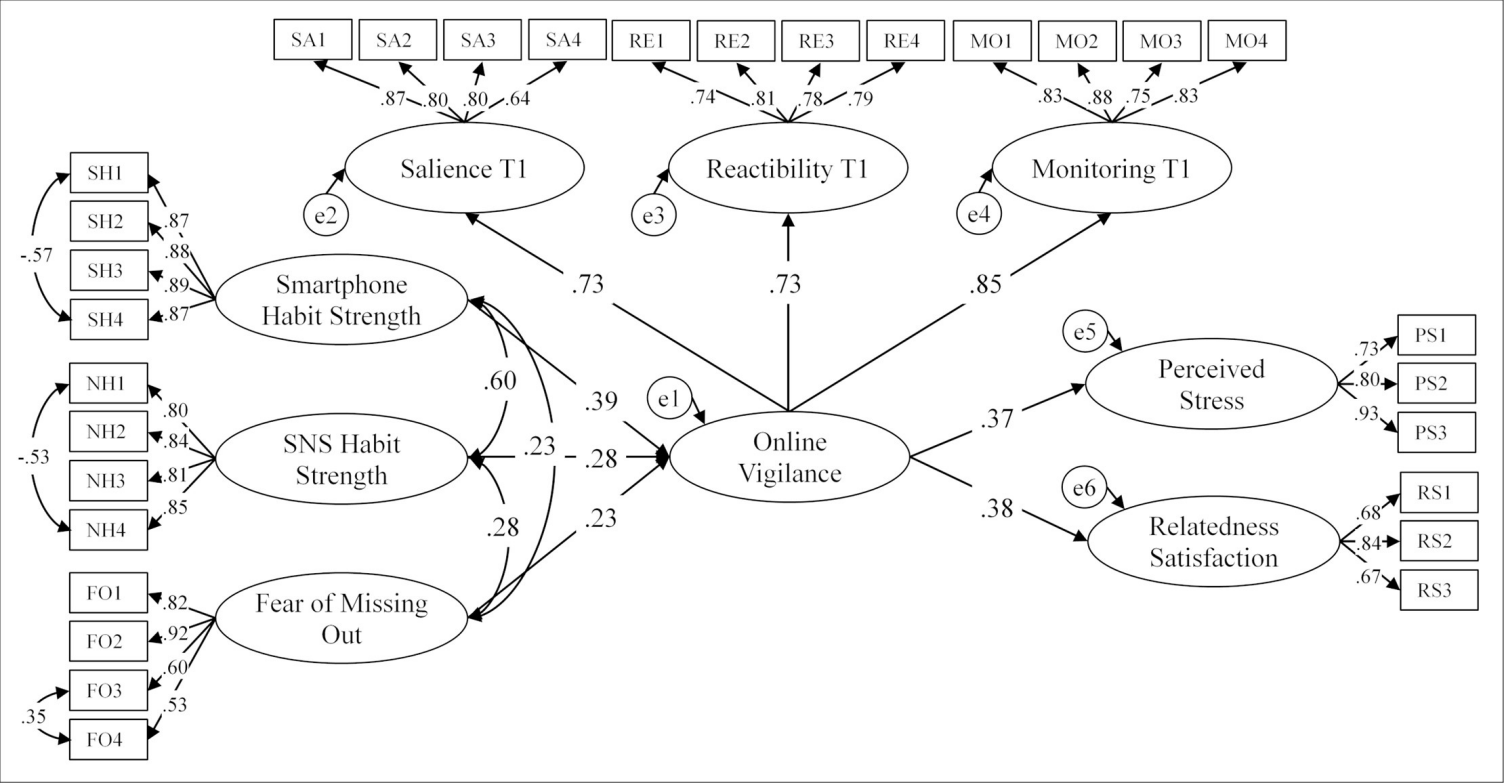
SEM assessing the construct validity of the Online Vigilance Scale. Based on data from *N* = 532 participants (Study 3), χ^2^(391) = 804.486, *p* < .001, CMIN/*df* = 2.06, CFI = .957, RMSEA = .045, 90% CI = [.040, .049], and SRMR = .052. Scores in the figure represent standardized path coefficients significant at *p* < .01. Relatedness satisfaction was measured with regard to interpersonal online communication.

We began our analysis by computing a structural equation model (SEM) using the AMOS 23 software and the maximum likelihood method. Our data did not meet the assumption of multivariate normality. We thus bootstrapped the significance of all model coefficients, using the same bootstrapping method as in Study 2. All significant statistical relationships reported below were confirmed with the bootstraping method. The SEM included online vigilance as a second-order factor with the three sub-dimensions of salience, reactibility, and monitoring as first-order factors (cf. Study 2). To assess construct validity, we used the T1 (Monday) assessment of online vigilance (*N* = 532), as the T2 (Friday) data has a lower power due to attrition (*N* = 448). FOMO, smartphone habit strength, and SNS habit strength were entered as exogenous variables predicting the second-order online vigilance factor and were allowed to covary. During model specification, it became apparent that the measurement model of each of the three exogenous variables required the inclusion of a covariance between error terms. These three covariances were included as well. Perceived stress (aggregated at person-level) and relatedness need satisfaction in online communication were entered as outcomes of online vigilance. The model (Fig 2) showed an acceptable fit to the data with χ^2^(391) = 804.486, *p* < .001, CMIN/*df* = 2.06, CFI = .957, RMSEA = .045, 90% CI = [.040, .049], and SRMR = .052. All three predictors were positively and significantly related to the second-order online vigilance construct (.23 ≤ β ≥ .39, all *p* < .01) and together explained 49% of its variance. Online vigilance, in turn, positively predicted perceived stress (β = .37, *p* < .001) as well as relatedness need satisfaction in online communication (β = .38, *p* < .001) and explained 13% and 14%, respectively, of each construct’s variance.

**Fig 3:**
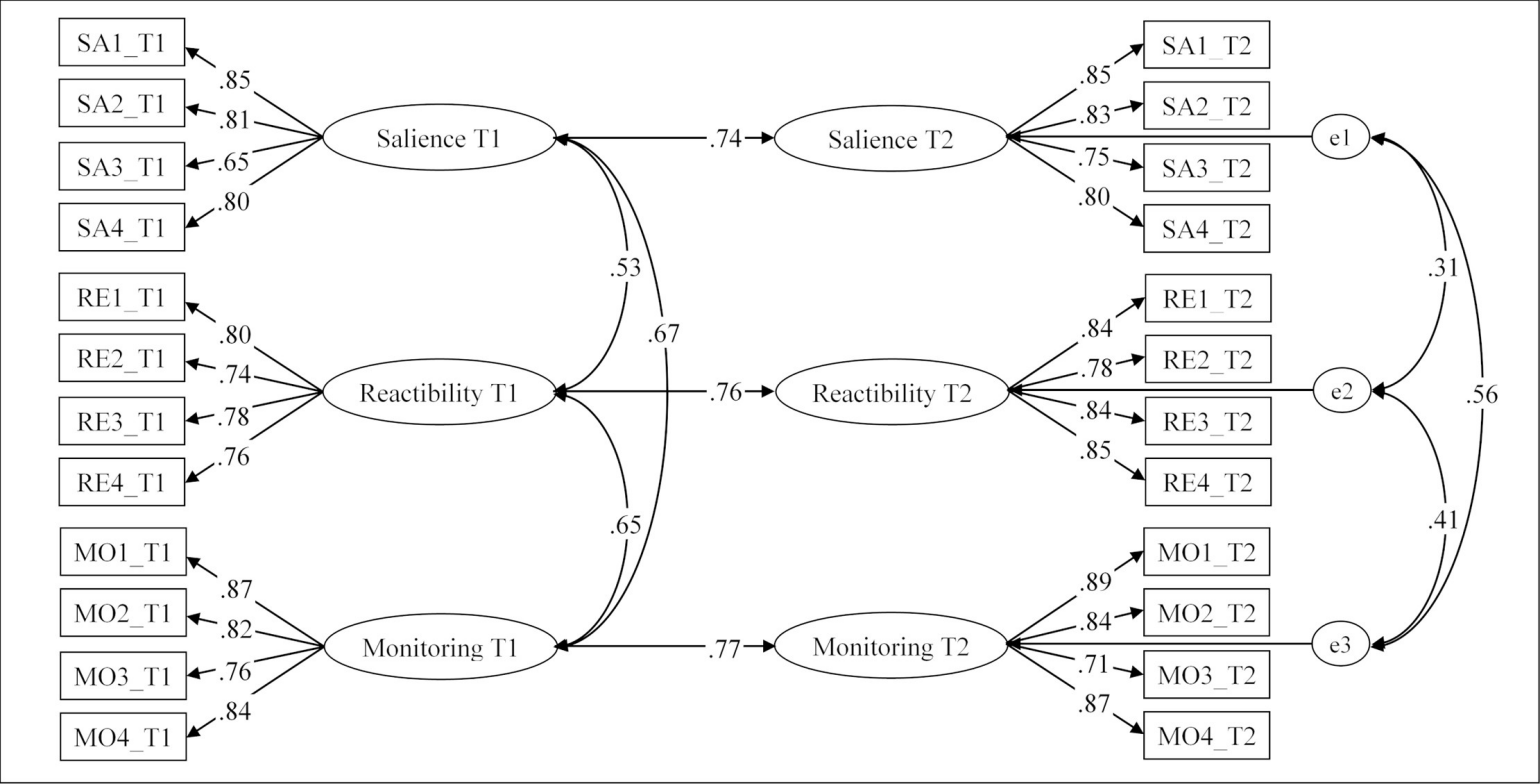
SEM estimating the test-retest reliability of the Online Vigilance Scale. Based on data from *N* = 448 participants (Study 3) who completed both the T1 (Monday) and T2 (Friday) assessments of the scale, *χ*^2^(243) = 597.86, *p* < .001, CMIN/*df* = 2.46, CFI = .953, RMSEA = .057, 90% CI = [.051, .063], and SRMR = .055. Scores in the figure represent standardized path coefficients significant at *p* < .001.

We went on to evaluate the test-retest reliability of the three dimensions of online vigilance and computed a second SEM with the maximum likelihood method in AMOS. The 12 items of the Online Vigilance Scale assessed at T1 and T2, respectively, were used to estimate the latent constructs of salience, reactibility, and monitoring at both points of measurement. The analysis was thus based on data from *N* = 448 participants who completed both the T1 (Monday) and T2 (Friday) assessments of the scale. Each latent construct measured at T1 was used to predict its respective counterpart measured at T2. The three sub-dimensions measured at T1 and the error terms of the three sub-dimensions measured at T2 were allowed to covary in the model (see Fig 3).

With *χ*^2^(243) = 597.86, *p* < .001, CMIN/*df* = 2.46, CFI = .953, RMSEA = .057, 90% CI = [.051, .063], and SRMR = .055, the model showed an acceptable fit to the data. All 12 items of the Online Vigilance Scale showed high factor loadings on their respective subscales (≥ .65). Furthermore, all sub-dimensions measured at T1 were strong predictors of the respective counterparts measured at T2 (all βs ≥ .74, all *p*s < .001).

### Discussion

The contribution of Study 3 is threefold: First, the factor structure and internal consistency of the Online Vigilance Scale was confirmed once more in an independent sample and over two points of measurement. Second, our SEM analysis (Fig 2) provides preliminary evidence for both the convergent as well as the discriminant validity of the Online Vigilance Scale. As predicted, the scale showed significant positive correlations with two related, but distinct theoretical constructs (smartphone and SNS habit strength), as well as an important precursor (fear of missing out) and two outcomes (perceived stress and relatedness need satisfaction in online communication) of online vigilance. Crucially, the results demonstrate that online vigilance shows only moderate empirical overlap with device- or channel-specific habit strengths (here: smartphone and SNS habit strengths), underlining the discriminant validity of the scale. Moreover, our results underline the complexity of the online vigilance construct as well as a key difference from Internet addiction: Online vigilance was positively associated with *both* a variable indicating negative effects of Internet use (stress) *and* a variable underlining its contribution to human functioning (relatedness need satisfaction in online communication). Thus, results show that online vigilance seems to have both a dysfunctional and a functional side. Yet the negative effects in form of stress assessed in the present study appear relatively mild in comparison to the severe functional impairments that define Internet addiction [59]. In contrast, the positive effects of online vigilance revealed in Study 3 provide first, albeit indirect support for the discriminant validity of the scale.

Finally, Study 3 demonstrates that the scale has a high test-retest reliability. This has important methodological implications as it suggests that the scale can be reliably used for research designs with multiple points of measurement (e.g., longitudinal surveys). Furthermore, the high temporal consistency of the measure also supports the notion that online vigilance represents a relatively stable individual predisposition for a POPC mindset. This mindset seems to show temporal stability despite the relatively high volatility of POPC behavior.

## Study 4: Validation using the day reconstruction method

In addition to the temporal *stability* demonstrated in Study 3, another important factor in the conceptualization and measurement of online vigilance is the aspect of *permanence* in the users‘ mindset: Users who are truly permanently online and permanently connected should also score higher in *situational* measures of online vigilance compared to users who are only at certain times highly engaged with their smartphone and their online sphere, or who do not hold cognitive routines of frequent online connection at all. In other words, our *person-level* measure of an online vigilance disposition should be a significant predictor of *state* variations in online vigilance. In order to validate the developed scale further, Study 4 was designed to identify the instrument’s capability to reflect individual differences in state online vigilance over the course of a ‘normal’ day.

### Method

#### Sample and procedure

A sample of *N* = 244 university students (52% female, *M*_age_ = 23.26 years, *SD* = 3.17) residing in a large city in Northern Germany participated in an online survey on “media use”. Calls for participation were circulated electronically through university-run online forums and printed posters displayed in diverse university facilities. A voucher of a major online shopping website with a value of 10 EUR was offered for completion of the survey. Informed consent was obtained with the same procedure used in Study 1, Study 2, and Study 3.

The day reconstruction method (DRM) was applied to validate the Online Vigilance Scale. The DRM has been used in psychological research to cover various aspects of people’s behavior, cognitions, affect, and experiences with high temporal resolution, which is achieved by assisting respondents in creating structured memories of the previous day [84]. Participants are first asked to divide the activities of the preceding day into a sequence of episodes. Respondents are then asked to respond to questions of interest for each of these segments. In the present study, participants responded to single-item measures of *salience* (“I was constantly thinking about what was happening online, even when I was not using my smartphone”), *reactibility* (“When I received an online message, I immediately gave it my full attention”), and *monitoring* (“I was constantly monitoring on my smartphone what was happening online”) for each of the time partitions they had defined. These single items were adapted from the respective subscale of the trait Online Vigilance Scale and their wording was adjusted to better fit the state character of the DRM measure. Participants responded to these items on a scale from 1 “does not apply at all” to 5 “fully applies”. The salience item was assessed in all episodes, whereas the other two items were only presented to participants for those episodes in which they reported having looked at their smartphone at least once. It is important to note that Study 4 did not aim at the development and validation of an independent state-level measure of online vigilance. The three items used in the DRM represent a pragmatic attempt to provide preliminary single-item state measures to enable a first test of the relationship between person-level and situation-level online vigilance. The development and validation of a more comprehensive measure of state online vigilance remains an important task for future research.

The DRM data were weighted by the duration of each time segment so that scores entered for longer time units had proportionally greater influence on the aggregated score for the day than entries from shorter segments [84]. On average, participants structured the reference day into 9.80 episodes (*SD* = 4.34).

Prior to completing the DRM, participants responded to the 12 items of the Online Vigilance Scale as used in the previous studies and provided demographic information. All three sub-dimensions of online vigilance showed satisfactory internal consistency (Salience: *α* = .83; Reactibility: *α* = .76; Monitoring: *α* = .83).

### Results

As a first step, Pearson correlations were computed to test whether the person-level values measured with the Online Vigilance Scale were significantly reflected in DRM data. The results indicate consistent and substantial positive associations between person-level and aggregated DRM values (weighted by episode duration, see Table 5).

**Table 5:** Correlations between person-level and situation-level measures of online vigilance (Study 4).

	Person-Level Measures			
Situation-Level (DRM) Measures	*M* (*SD*)	Salience	Reactibility	Monitoring
*M* (*SD*)		1.81 (0.77)	2.64 (0.80)	2.52 (0.95)
Salience	1.56 (0.69)	.47***	.30***	.42***
Reactibility	2.73 (0.95)	.20**	.36***	.30***
Monitoring	1.98 (0.83)	.47***	.40***	.42***

*Note*. Based on *N* = 236 participants. Situation-level measures were aggregated at person level and duration-weighted.

** *p* < .01

*** *p* < .001.

To further assess how each of the dimensions of person-level online vigilance predicts state-level vigilance and how much variance of state-level vigilance can be explained by between-person differences, we calculated a series of multilevel random intercept regression models using R package lme4 [85]. The three vigilance dimensions measured at person level were centered on their respective sample mean (“grand mean centering”). Results (see Table 6) overall support our assumption that online vigilance substantially predicts situational variation in online vigilance. All three dimensions showed high intraclass correlation coefficients (ICCs) between .30 and .53, indicating that between-person differences accounted for a large portion of the variance on the three situation-level vigilance measures. Person-level salience, reactibility, and monitoring together explained between 26% and 38% of this between-person variance (see *Pseudo-R*^*2*^ values based on the formula by Snijders and Bosker [86] in Table 6). As expected, situation-level salience and reactibility were each best predicted by their person-level counterparts. Situation-level monitoring, however, was most strongly predicted by person-level salience.

**Table 6:** Random intercept regressions predicting situation-level online vigilance from grand-mean centered person-level online vigilance (Study 4).

		Salience		Reactibility		Monitoring	
		Model 0	Model 1	Model 0	Model 1	Model 0	Model 1
		*b* (se)	*b* (se)	*b* (se)	*b* (se)	*b* (se)	*b* (se)
Intercept		1.67*** (0.05)	1.69*** (0.04)	2.88*** (0.06)	2.91*** (0.05)	2.08*** (0.05)	2.10*** (0.04)
Person-level predictors							
	Salience	—	0.39*** (0.07)	—	0.01 (0.08)	—	0.31*** (0.07)
	Reactibility	—	0.06 (0.06)	—	0.40** (0.08)	—	0.17** (0.07)
	Monitoring	—	0.16** (0.06)	—	0.12 (0.07)	—	0.15* (0.06)
Goodness-of-fit statistics							
	AIC	2932	2853	3990	3948	3776	3707
	Log likelihood	-1463	-1420	-1992	-1968	-1885	-1848
	Deviance	2926	2841	3984	3936	3770	3695
	Δ Deviance (χ^2^)	—	85.6	—	47.6	—	74.7
	*df*	—	3	—	3	—	3
	*p*	—	< .001	—	< .001	—	< .001
Intraclass correlation coefficient (ICC)		.53	—	.33	—	.30	—
Between-person variance explained *(Pseudo-R*^*2*^*)*		—	.36	—	.26	—	.38

*Note*. Based on data from *N*_Participants_ = 236 and *N*_Situations_ = 1262; depicted are unstandardized linear coefficients with standard errors in parentheses; estimation method: maximum likelihood; significance tests for fixed effects: t-tests with Satterthwaite's approximation of *df*; person level predictors were centered on their sample mean (“grand mean”).

*** *p* < .001

** *p* < .01

* *p* < .05.

### Discussion

The correlational patterns obtained from using the DRM contribute to the successful validation of the developed scale for online vigilance with regard to the aspect of ‘permanence’. Higher person-level scores in (trait) online vigilance correspond with more frequent situation-level occurrences of salience, reactibility, and monitoring. Hence, Study 4 extends the previous validation of the scale based on DRM data with high temporal resolution. The strength of the obtained correlations suggests, however, that for many users and/or many parts of a normal day, online vigilance is not so much ‘truly permanent’, but that there are substantial intra-individual variations in (state) online vigilance over the day. While this points to the importance to study such temporal dynamics in future research, it does not put the validity of the scale into question: In addition to the high consistency and reliability shown in Studies 1 to 3, Study 4 demonstrates that the instrument is capable of differentiating clearly between users with different degrees of person-level online vigilance, as it substantially explains individual differences in situation-level online vigilance.

## General discussion

Using mobile Internet devices and accessing relevant others, media, and services online anytime and anywhere has become ‘natural’ for many people across the globe. With online vigilance, we propose a new concept that captures the cognitive, attentional, and motivational tendencies that frequent and multi-purpose use of mobile online devices is likely to bring about. *Salience* of the online world, *reactibility* to communication dynamics emerging from one’s online sphere, and *monitoring* of the online environment are the dimensions that jointly describe the individual predispositions shaped by the POPC environment [8]. Based on four studies with different samples and methodologies, a robust, validated, reliable, and economical self-report measure of this communicational disposition is now available. In combination, the four studies suggest that the Online Vigilance Scale (OVS) shows a stable factor structure in various contexts and user populations (Studies 1–3). Our findings clearly support the notion of online vigilance as an individual difference variable with considerable temporal stability (Study 3) and the ability to explain variance in state measures of online vigilance (Study 4).

Beyond its reliability and psychometric properties, the present research also provides evidence of the construct validity of the OVS. In combination, Study 2 and Study 3 provide a nomological network of a selection of related concept, predictors, and outcomes of online vigilance. The results of Study 2 not only demonstrate, that online vigilance shows the expected positive relationship with various measures of Internet use, but also predicts how online media are integrated into and are intertwined with offline activities (i.e., Internet multitasking). The results of Study 3 suggest that the scale shows both convergent and discriminant validity: the scale significantly correlated with plausible predictors (i.e., fear of missing out) and outcomes (i.e., perceived stress) of online vigilance in the predicted directions. The moderate correlations between the OVS and smartphone as well as SNS habits found in Study 3 are particularly relevant, as they suggest that online vigilance is related to, yet distinct from Internet habits, supporting our theoretical argumentation and concept explication. The fact that online vigilance showed similar correlations with two different forms of online media use habits (smartphone and SNS) further supports our theoretical conceptualization of online vigilance as a platform-independent cognitive orientation towards online content and communication. Finally, our data provide first *indirect* evidence for a conceptual distinction between online vigilance and Internet addiction. While severe functional impairment resulting from Internet use is a defining feature of behavioral addiction [59,60], the findings of Study 3 suggest that online vigilance–albeit being associated with milder negative outcomes such as stress–can also have beneficial effects, such as increased relatedness need satisfaction.

### Limitations and open questions

Although the four studies presented here justify considerable confidence in the psychometric properties and validity of the scale, a number of methodological limitations and open questions need to be taken into consideration. First, although the presented studies tested the Online Vigilance Scale in a number of heterogeneous samples, the invariance of the measure *between* different populations remains unclear. Future research should thus aim at sampling from different populations and cultural contexts within the same study to provide a basis for the test of group-invariance of the scale. Furthermore, while we have tested and replicated the factor structure and dimensionality of the Online Vigilance Scale in multiple studies and based on various forms of estimation (ML, Bayesian, bootstrapping), future research could further validate the structure and psychometric properties of the scale using additional statistical approaches such as item response theory.

Furthermore, the findings of all four studies are subject to the typical limitations of cross-sectional self-report data. The causal relationships and direction of effects between online vigilance and related constructs such as media habits or the fear of missing out (see Study 3) thus remain unclear. The use of longitudinal panel data in future research will be necessary to better understand the nomological network of online vigilance.

The constant connectedness, the ubiquity of connection cues, and the high number of short usage episodes (e.g., checking behavior) that are typical for Internet use in a POPC environment make it increasingly hard for survey respondents to provide accurate estimates of their Internet usage behavior [87]. Future research exploring the relationship between online vigilance and Internet use would thus benefit from using tracking methodology to provide an accurate measure of usage patterns.

While the present research provides preliminary evidence of the relationship between person-level and situation-level online vigilance, the day reconstruction method used in Study 4 requires participants to remember situational levels of online vigilance on the preceding day. This may come at the expense of data accuracy as some respondents may find it hard to reconstruct their levels of online vigilance in retrospect. Future research should thus replicate the findings of Study 4 by using mobile experience sampling methodology that allows for assessing online use and the accompanying affect and cognitions in situ (e.g., [20,88]).

A last methodological limitation refers to the wording of the items of the reactibility subscale of the Online Vigilance Scale. Rather than to the online sphere per se, these items refer to reacting to incoming *messages*. The subscale thus could be criticized for exclusively measuring a motivational predisposition to prioritize online messages rather than general online activities. We propose however, that online messages and the accompanying notifications are the most prevalent connection cues present in the POPC media environment [9,29], thus representing a particularly salient “anchor” for the instrumental and attentional learning processes that drive the development of online vigilance. We thus believe that the items provide a valid basis for the measurement of the reactibility sub-dimension of online vigilance. Should the technological affordances of online communication change and make messages a less salient connection cue, however, future research may have to adapt the wording of the respective items to better reflect the status quo of the POPC environment.

Besides these methodological limitations, the present findings also leave a number of open questions for future research. While Study 2 and 3 provide insights into the relationship between online vigilance and a number of different variables, the learning processes proposed to drive the formation of online vigilance remain untested. The same applies to the sources of individual differences in online vigilance discussed in the theory section. The positive correlation between the fear of missing out and online vigilance documented in Study 3 lends preliminary support to the notion of varying susceptibility to the learning mechanisms underlying online vigilance. It appears plausible to assume that the informational and social gratifications offered by social media are particularly valuable to individuals with high FOMO [53], presumably making them more susceptible to the acquisition of high levels of online vigilance. Without longitudinal data that would provide insight into the trajectory of the development of online vigilance, however, questions pertaining to the acquisition process and the intervening variables resulting in individual differences in online vigilance remain unanswered.

Open questions also remain with regard to the differentiation of online vigilance from related concepts. While the moderate correlations found between online vigilance and SNS as well as smartphone habits suggest that online vigilance goes significantly beyond habits, the specific relationship between both concepts remains unclear. It could be argued, for example, that SNS and smartphone habits represent relatively broad forms of gateway habits [89] and that application or content-specific habits, such as Facebook checking habits [63], may correlate more strongly with online vigilance. Furthermore, in our concept explication, we suggested that while habits exclusively refer to automated processes, online vigilance also integrates deliberate and controlled aspects. The data of the studies presented here, however, do not differentiate between deliberate and automated processes resulting from online vigilance and thus do not provide a test of this assumption. Overall, the relationship between online vigilance and habits needs to be further explored in future research.

The same applies to the conceptual differentiation of online vigilance and Internet addiction. While the positive relationship between online vigilance and relatedness need satisfaction found in Study 3 could be interpreted as a first indirect indicator of the conceptual distinctiveness from the exclusively dysfunctional consequence of addictive use, other interpretations are also plausible. Given the cross-sectional nature of the data, it is unclear, for example, whether relatedness need satisfaction is really a result of online vigilance or rather a precursor driving the development of higher levels of vigilance. Furthermore, the present research does not provide a test of the *direct* relationship between online vigilance and addiction. However, the recent criticism of using symptoms such as preoccupation or tolerance typically associated with substance-induced addictions to measure behavioral addiction [59,60], may call the usefulness of such a direct comparison into questions. What would it mean, for example, if the salience dimension of the Online Vigilance Scale would show a high positive correlation with the “symptom” of preoccupation with or distraction by Internet use frequently assessed in Internet addiction scales (e.g., [90])? Would this indicate a lack of discriminant validity of the Online Vigilance scale or suggest that preoccupation is a bad indicator of Internet addiction? Rather than attempting to contrast both concepts, future research might benefit more from considering online vigilance and Internet addiction as different points on a continuum from unproblematic to pathological use. From this perspective, the strong involvement with online communication associated with online vigilance could be a risk factor increasing the vulnerability for Internet addiction [58]. Thus, finding answers to the question of when and how online vigilance becomes problematic is an interesting challenge for future research.

### Conclusion

Overall, we believe that the four studies presented here clearly underline the reliability, validity, and usefulness of the Online Vigilance Scale. We are confident that the scale will be instrumental and provide new impulses in various contexts of research on Internet, mobile media use, and related social behavior. The scale may be useful to measure online vigilance as a *determinant* of relevant communication processes and outcomes. For instance, online vigilance may explain variance in audience selection of and responses to news, entertainment, or persuasive content that is disseminated through (mobile) social media. Online vigilance may also be measured as a *moderator* of media effects, since people high in online vigilance may respond differently from users low in online vigilance, for example, with regard to elaboration strength [91] or affective responses to breaking news [92]. The measure of online vigilance may also serve as a dependent variable, that is, online vigilance may be seen as a relevant *outcome* to be explained: Research on the adoption of communication innovations [93] or on developmental processes of media socialization [94] are just two examples of research areas that could benefit from addressing online vigilance as a relevant outcome variable in future studies.

Furthermore, we believe that the concept of online vigilance may provide new impulses for existing theory and theoretical models in the context of computer-mediated communication (CMC). Online vigilance may, for example, be an individual difference variable shaping the intensity and temporal dynamics of the selective self-presentation and impression formation processes in CMC addressed in the hyperpersonal model of interpersonal communication [95]. Online vigilance may also provide new impulses for theory and research addressing effects of self-presentation through the lens of self-affirmation or social comparison theory [96], suggesting that it may be a powerful predictor of the differential susceptibility to such effects. We further believe that online vigilance has important implications for interpersonal interactions in CMC. The concept could complement traditional theories in this context, such as expectancy violations theory [97], by identifying the cognitive and motivational effects driving the negotiation of and coping with interpersonal expectations in the POPC environment. Furthermore, online vigilance could provide impulses to more recent theoretical developments, such as the Communicate Bond Belong Theory [98], by complementing our understanding of the homeostasis of interpersonal needs via CMC. Finally, despite the remaining open questions regarding the direct relationship between online vigilance and Internet addiction, we believe that our theoretical approach may provide new opportunities for a differentiated view on strong involvement with online content and a potential alternative to “overpathologizing everyday life” [59].

In sum, the Online Vigilance Scale offers a powerful and versatile expansion of the toolkit of empirical social science for many of those challenges that the continuing online revolution is bringing about. It can assist researchers in responding to the rapid diffusion of mobile online communication whenever a user-centered perspective is of research interest and may help explain how and why technological change is affecting the minds and communication behaviors of so many people every day.

## Supporting information

S1 Dataset Study 1. (SAV)

S2 Dataset Study 2. (SAV)

S3 Dataset Study 3. (SAV)

S4 Dataset Study 4. (SAV)

S5 Dataset Study 4 wide format. (SAV)
